# Adjuvant effect of IRES-based single-stranded RNA on melanoma immunotherapy

**DOI:** 10.1186/s12885-022-10140-2

**Published:** 2022-10-05

**Authors:** Hye Won Kwak, So-Hee Hong, Hyo-Jung Park, Hyeong-Jun Park, Yoo-Jin Bang, Jae-Yong Kim, Yu-Sun Lee, Seo-Hyeon Bae, Hyunho Yoon, Jae-Hwan Nam

**Affiliations:** 1 Department of Medical and Biological Sciences, The Catholic University of Korea, 43-1 Yeokgok-dong, Wonmi-gu, Bucheon, 14662 Republic of Korea; 2 BK Plus Department of Biotechnology, The Catholic University of Korea, Gyeonggi-do, Bucheon, Republic of Korea; 3 SML biopharm, Gyeonggi-do, Gwangmyeong, Republic of Korea; 4 Department of Microbiology, College of Medicine, Ewha Womans University, Seoul, 07804 Republic of Korea

**Keywords:** Murine melanoma model, Adjuvant immunotherapy, CrPV^IRES^-ssRNA, RNA vaccine, Antigen-presenting cells

## Abstract

**Background:**

Adjuvant therapies such as radiation therapy, chemotherapy, and immunotherapy are usually given after cancer surgery to improve the survival of cancer patients. However, despite advances in several adjuvant therapies, they are still limited in the prevention of recurrences.

**Methods:**

We evaluated the immunological effects of RNA-based adjuvants in a murine melanoma model. Single-stranded RNA (ssRNA) were constructed based on the cricket paralysis virus (CrPV) internal ribosome entry site (IRES). Populations of immune cells in bone marrow cells and lymph node cells following immunization with CrPV^IRES^-ssRNA were determined using flow cytometry. Activated cytokine levels were measured using ELISA and ELISpot. The tumor protection efficacy of CrPV^IRES^-ssRNA was analyzed based on any reduction in tumor size or weight, and overall survival.

**Results:**

CrPV^IRES^-ssRNA treatment stimulated antigen-presenting cells in the drain lymph nodes associated with activated antigen-specific dendritic cells. Next, we evaluated the expression of CD40, CD86, and XCR1, showing that immunization with CrPV^IRES^-ssRNA enhanced antigen presentation by CD8a^+^ conventional dendritic cell 1 (cDC1), as well as activated antigen-specific CD8 T cells. In addition, CrPV^IRES^-ssRNA treatment markedly increased the frequency of antigen-specific CD8 T cells and interferon-gamma (IFN-γ) producing cells, which promoted immune responses and reduced tumor burden in melanoma-bearing mice.

**Conclusions:**

This study provides evidence that the CrPV^IRES^-ssRNA adjuvant has potential for use in therapeutic cancer vaccines. Moreover, CrPV^IRES^-ssRNA possesses protective effects on various cancer cell models.

**Supplementary Information:**

The online version contains supplementary material available at 10.1186/s12885-022-10140-2.

## Introduction

The ideal vaccine protects against infection by inducing a long-term immunological memory response to the target antigen [1]. However, some vaccines can fail to elicit a sufficient immune response and, therefore, cannot protect against infection or disease recurrence. Various immune stimulants, namely adjuvants, have been developed that increase both innate and humoral responses [2]. Immune stimulation can generally be achieved through three signaling mechanisms [3, 4]: 1) Increased antigen presentation and antigen uptake [5–7] by allowing the interaction of pattern of recognition receptors (PRRs) such as Toll-like receptors (TLRs), retinoic acid-inducible gene I (RIG-I)-like receptors (RIG-Is) & NOD-like receptors, with pattern associated molecular pathogens [8–11]; 2) Activation of the co-stimulatory molecules of antigen-presenting cells (APCs), such as CD40, CD80, and CD86 cells [11, 12]; 3) Stimulation of naïve T cells to differentiate into Th1/Th2 cells and the modulation of antigen-specific antibody responses via cytokine activation and PRR stimulation [13–16].

Aluminum (alum)-containing supplements act as exogenous damage-associated molecular patterns (DAMPs), triggering an immune response and causing tissue damage [17]. Aluminum exposure has been shown to induce antibody-mediated responses and the proliferation of CD4 T cells [18]. Alum combined with antigenic proteins enhanced the differentiation of Th2 cells and increased antigen-specific follicular T cells [14]. On the other hand, nucleic acid-based adjuvants generally induce an immune response through the recognition of foreign agents and non-self-nucleic acids through TLRs located on the surface of APCs (TLR1, 2, 4, 5, and 6) and endosomal membranes (TLR3, 7, 8, and 9) [19]. RNA-based adjuvants have been reported to recognize a variety of TLRs, such as TLR3 for dsRNA and TLR7/8 for ssRNA [20, 21]. Poly(I:C) consists of double-stranded RNA, which generally induces antiviral effects by stimulating TLR3 signaling [22]. It also enhances type I interferon and Th1-related cytokines [13, 23], and subsequently activates co-stimulatory molecules on conventional dendritic cells (cDCs), which affects CD8 T cell activation [24–26]. However, poly(I:C) has significant drawbacks, including severe side effects, an undefined structure, and a lack of homogeneity [27]. In contrast to poly(I:C), synthetic or natural ssRNA is recognized by TLR7/8 in plasmacytoid dendritic cells (pDCs) which in turn produce type I interferon and proinflammatory cytokines [24, 25]. Activated TLR7/8 may also promote the activation of CD8 and effector T cells [26, 28].

Recently, an ssRNA-based adjuvant, RNA adjuvant®, has been developed. It consists of an ssRNA, a non-coding site, and non-capped RNA sequence containing with poly (U) repeats, with added cationic peptides for stabilization. RNA adjuvant® therapy has been shown to enhance antigen-specific antibody responses and balanced Th1/Th2 cell responses [29]. It also induced immune responses via TLRs, RIG signaling, and MyD88-/ MAVS-dependent mechanisms [29, 30]. The induction of these signaling pathways contributes to the activation of human CD141^+^ DCs, pDCs, monocytes, and CD8 T cells [31–33]. RNA adjuvant® has been incorporated into various types of vaccine delivery systems, including peptide, subunit, and virus-like particle vaccines to induce immune responses similar to poly(I:C) adjuvant [29, 34]. Our group has previously reported that ssRNA-based adjuvant treatment effectively increased the efficacy of various prophylactic vaccines, including inactivated, subunit, virus-like particle, and even live-attenuated vaccines by inducing balanced Th1/Th2 cell responses [35–37].

An adjuvant can be used to enhance the immune response of a therapeutic vaccine. The RNA-based adjuvant increased the antibody response, which is an indicator of efficacy for the preventive vaccine, and also increased the antigen-specific T cell anti-tumor immune response [34]. In this study, the cricket paralysis virus internal ribosome entry site (CrPV^IRES^)-ssRNA was established and administered to mice challenged with murine melanoma cells. The National Center for Health Statistics reported that approximately 90,000 cases of melanoma occur annually in the USA [38]. Melanoma is the most common type of skin cancer, which occurs in melanocytes that produce the melanin responsible for variations in skin color [39]. Melanin is most abundant in the epidermis but is also found in the irises, inside the nose or throat, and throughout the internal organs [40]. Although the etiology of all types of melanomas is not currently elucidated, it is known that sun-induced ultraviolet (UV) radiation promotes the growth of melanoma [41]. Most early-stage melanomas can be treated successfully with surgery; however, this is often combined with chemotherapy or targeted therapy for metastatic melanoma [39]. Unfortunately, the survival rate of patients is still low due to recurrence after treatment, and thus, there is an urgent need for better strategies to treat melanoma. Here, we evaluate the effects of CrPV^IRES^-ssRNA as an adjuvant therapy that can induce antigen-specific T cell responses. In addition, we identify the immunological mechanisms of CrPV^IRES^-ssRNA activity and investigate the efficacy of CrPV^IRES^-ssRNA as an adjuvant in melanoma treatment.

## Material and methods

### Mice and immunization

Six-week-old female C57BL/6 mice were purchased from Dae-Han Bio-Link (Eumseong, Korea). Mice were maintained under specific pathogen-free conditions with a 12 h light/dark cycle. According to the National Institutes of Health (NIH) guidelines, euthanasia of rodents was performed using carbon dioxide at a fill rate of 30–70% of the chamber volume per minute. All animal studies were conducted according to the guidelines of the Institutional Animal Care at the Catholic University of Korea (CUK-IACUC-2021–025-01).

**Fig. 1:**
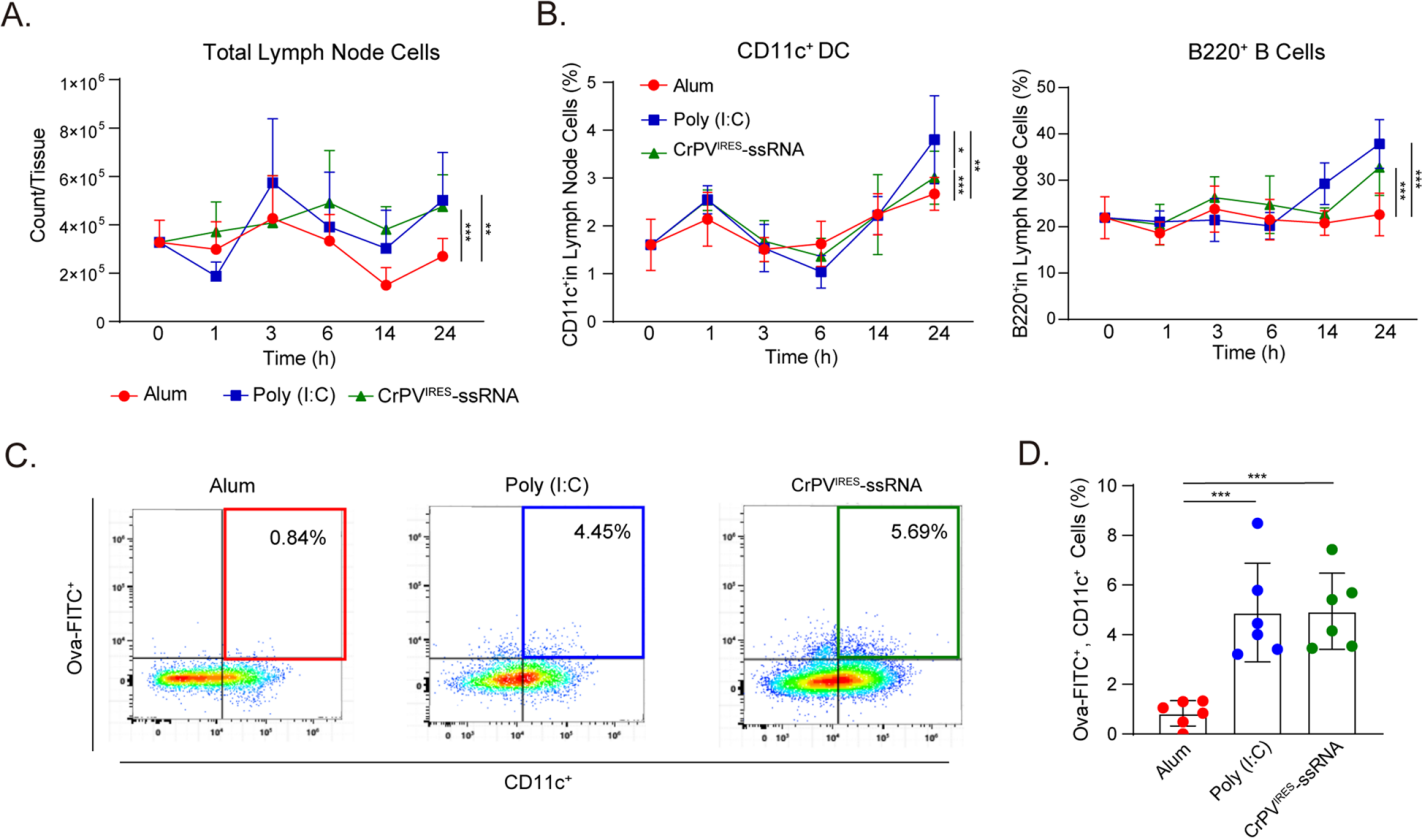
CrPV^IRES^-ssRNA increases the number of antigen-presenting cells. C57BL/6 mice were intramuscularly injected with CrPV^IRES^-ssRNA, alum, and poly(I:C). Immune cells were analyzed in drain inguinal lymph nodes for 24 h. **A** Total number of cells in draining inguinal lymph nodes according to adjuvant (*n* = 3). **B** Total cell percentages of CD11c^+^ DCs (left) and B220^+^ B cells (right). **C**, **D** Efficacy of antigen uptake using FITC-conjugated Ova 12 h after immunization (*n* = 6). Quantitative plots (**C**) and graph (**D**) of flow cytometry. Data were represented as mean ± SD. Statistical significance was indicated by *p < 0.05, **p < 0.01, and ***p < 0.005. Ova: Ovalbumin

**Fig. 2:**
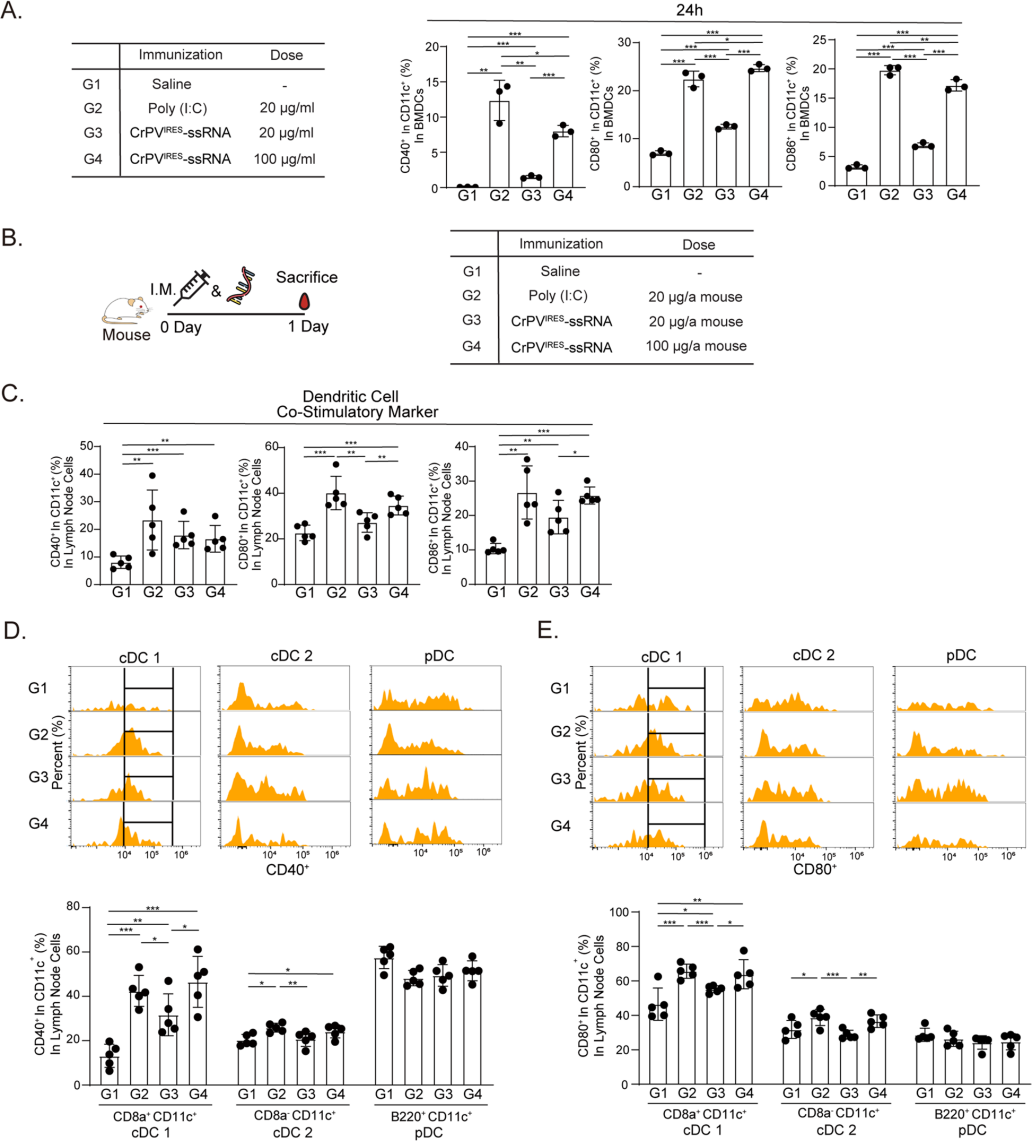
CrPV^IRES^-ssRNA activates dendritic cells in drain inguinal lymph nodes. **A** Mouse bone marrow-derived dendritic cells (BMDCs) were stimulated with CrPV^IRES^-ssRNA or poly(I:C) for 24 h. Cell activation was measured by flow cytometry using antibodies specific to CD40, CD80, and CD86. **B** Mice were intramuscularly immunized once with poly(I:C) (20 µg) and CrPV^IRES^-ssRNA (20 µg and 100 µg) for 24 h (*n* = 5). **C** Percentages of co-stimulatory molecules (CD40, CD80, and CD86) of CD11c^+^ DCs in lymphocytes isolated from drain inguinal lymph nodes. **D**, **E** Percentages of activation markers of DCs (cDC1, cDC2, pDC) in CD40 + cells (**D**) and CD80 + cells (**E**). Dendritic subsets were analyzed by flow cytometry using CD8a^+^, CD8^−^, and B220.^+^. Data were represented as mean ± SD. Statistical significance was indicated by *p < 0.05, **p < 0.01, and ***p < 0.005

**Fig. 3:**
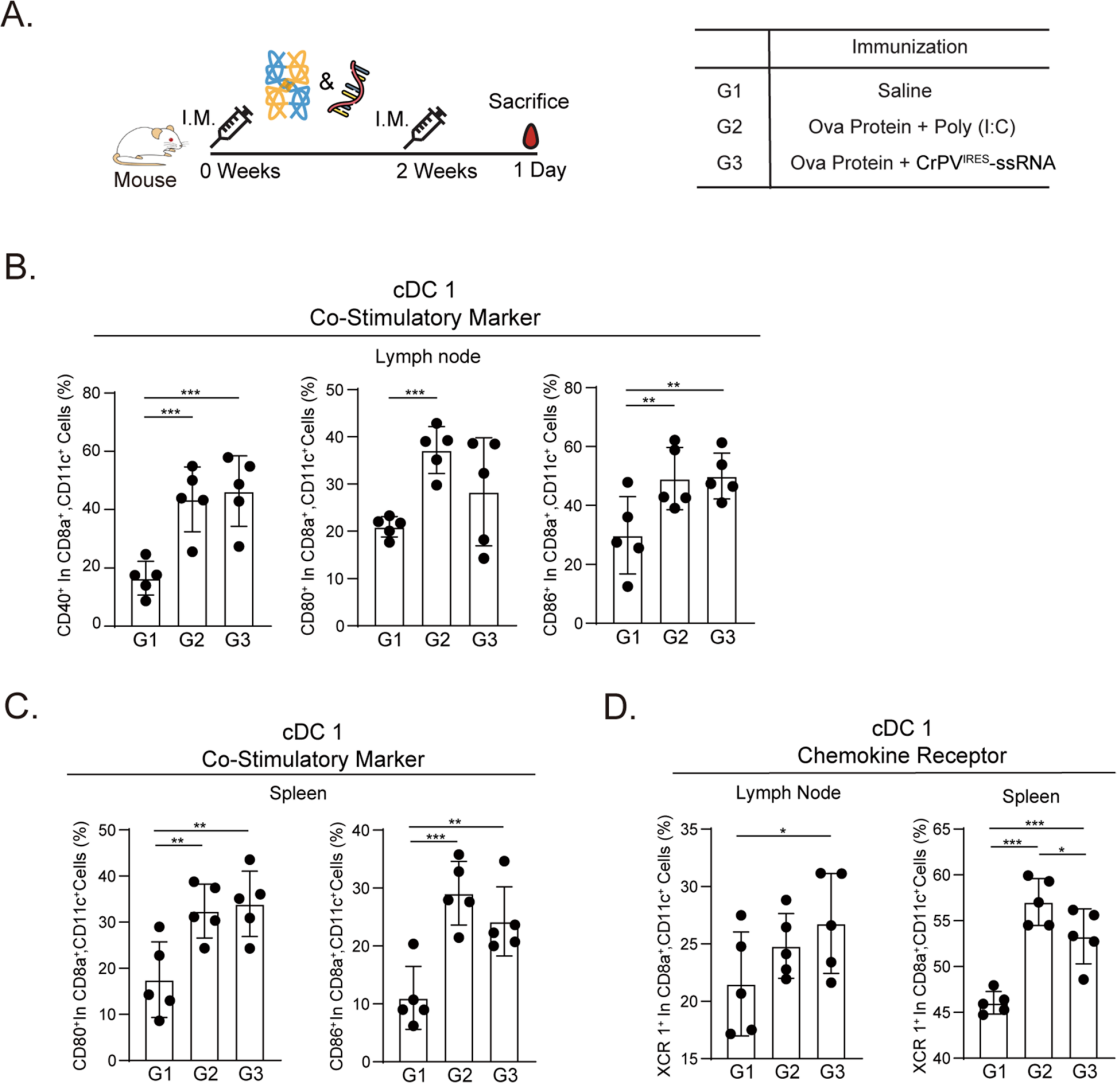
Ova protein enhances CrPV^IRES^-ssRNA-mediated conventional DC 1 activation. **A** The mice were primed and boosted with Ova protein (50 µg) formulated with CrPV^IRES^-ssRNA (20 µg) or poly(I:C) (20 µg) (*n* = 5). Splenocytes and drain inguinal lymph nodes were harvested 1 day after boosting. **B** Frequency of co-stimulatory CD40, CD80, and CD86 of CD8^+^ cDC1 cells. **C**, **D** XCR1 expression of CD8.^+^ cDC1 was measured in splenocytes (**C**) and lymphocytes (**D**) isolated from drain inguinal lymph nodes. Data were represented as mean ± SD. Statistical significance was indicated by *p < 0.05, **p < 0.01, and ***p < 0.005

**Fig. 4:**
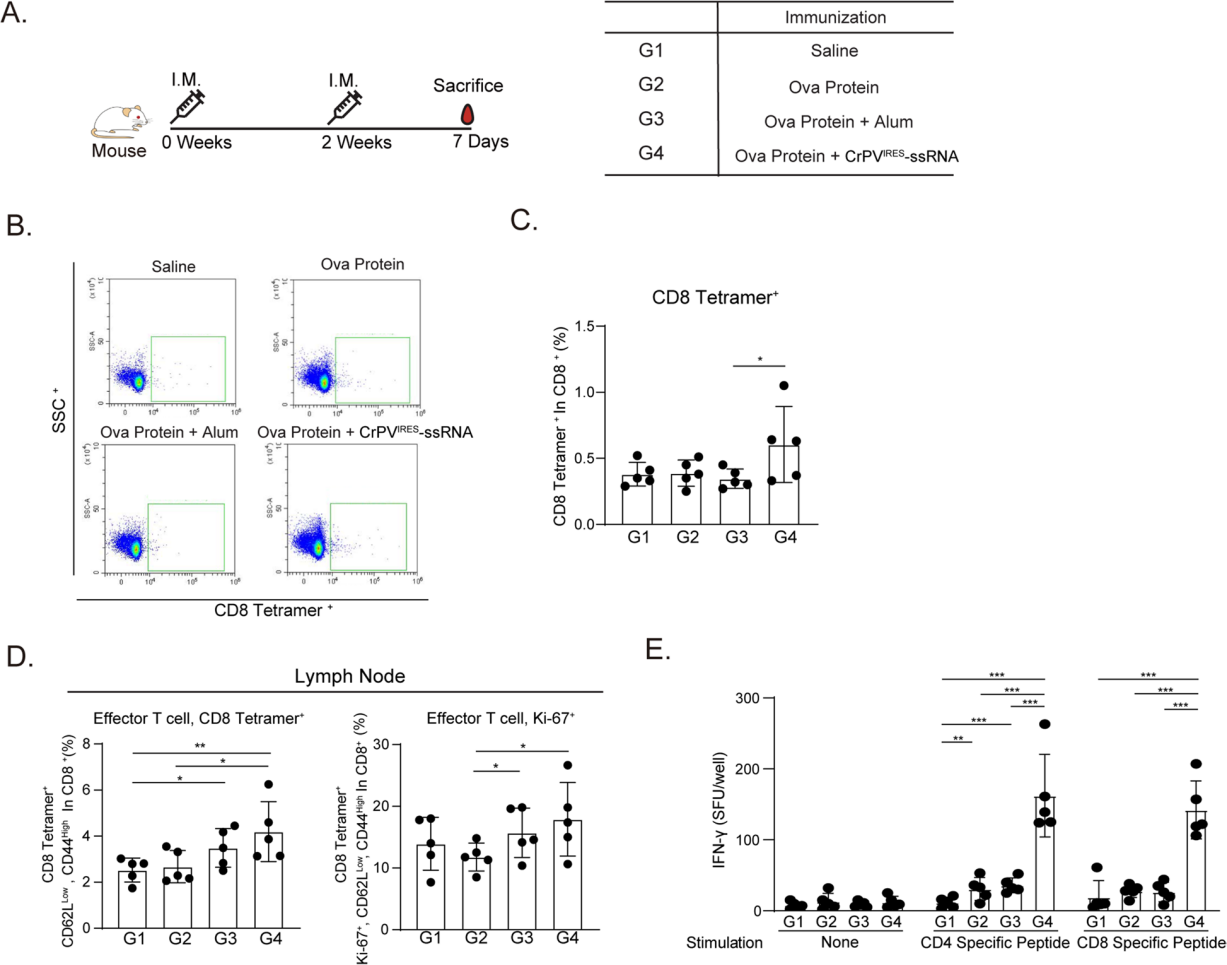
CrPV^IRES^-ssRNA conjugated with Ova protein induces antigen-specific CD8 T cell activation. **A** Mice (C57BL/6) were intramuscularly immunized twice at an interval of 2 weeks with Ova protein (50 µg) mixed alum (antigen ratio 1:1) or CrPV^IRES^-ssRNA (20 µg) (*n* = 5). Splenocytes and drain inguinal lymph nodes were harvested 7 days after boosting. **B**, **C** Ova-specific CD8 T cells were assessed using Ova 257–264 tetramer staining in splenocytes. Quantitative plots (**B**) and graph (**C**) of flow cytometry. **D** Frequency of effector T cells and Ki-67 were measured in inguinal lymph nodes. **E** ELISpot assay for IFN-γ secretion. Splenocytes were stimulated for 2 days with/without Ova-specific CD4 or CD8 T cell peptide. IFN-γ-producing cells were measured using ELISpot assay. Data were represented as mean ± SD. Statistical significance was indicated by *p < 0.05, **p < 0.01, and ***p < 0.005. ELISpot: enzyme-linked immunospot, IFN: interferon

To evaluate adjuvant therapy APC response in relation to total lymph node cells, mice were immunized intramuscularly with either Alum antigen in a 1:1 ratio (Thermo Fisher Scientific, Waltham, MA, USA), 20 µg poly(I:C) (Sigma Aldrich, St. Louis, MO, USA), or 20 µg ssRNA (*n* = 3) (Fig. 1). In the APCs activation test, mice were immunized intramuscularly with either saline, 20 µg poly(I:C), 20 µg ssRNA, or 100 µg ssRNA (Fig. 2). To test antigen-specific responses to Ova protein, mice were immunized intramuscularly twice with an interval of two weeks (day 0 and on day 14) with 50 µg Ova protein (Sigma Aldrich) in combination with either 20 µg poly(I:C) or 20 µg ssRNA. Saline injection was used as a control (Fig. 3). In the T-cell activation test, mice were immunized intramuscularly twice with an interval of two weeks (day 0 and on day 14) with either 50 µg Ova protein in isolation, 50 µg Ova protein with Alum antigen in a 1:1 ratio or 50 µg Ova protein with 20 µg ssRNA (Fig. 4).

### In vitro transcription and RNA purification

The DNA platform was constructed based on the IRES intergenic region and the SV40 late-polyadenylation signal sequences. DNA templates were linearized using Not I (Enzynomics, Daejeon, Korea). For ssRNA production, in vitro transcription was assessed using the EZ T7 High Yield In Vitro Transcription Kit (Enzynomics), according to the manufacturer’s instructions.

### Flow cytometry and antibodies

To confirm the presence of the OVA tetramer, splenocytes and the inguinal lymph node lymphocytes of the mice were harvested and blocked using CD16/CD32 (eBioscience, Waltham, MA, USA; Thermo Fisher Scientific Inc.) for 20 min at 4 °C, and then stained with the following antibodies for 20 min at 4 °C: H-2 Kb Ova tetramer with the Ova 257–264 epitope SIINFEKL. This was provided by the National Institutes of Health (NIH), and we followed the manufacturers’ protocol.

For surface staining, splenocytes and isolated immune cells from muscle and the inguinal lymph node were stained with the following antibodies for 15 min; CD8a (clone KT15, MBL), Fixable Viability Dye eFluor520 (eBioscience), CD8 (Clone 53–6.7, Invitrogen Waltham, MA, USA), CD44 (Clone IM7, Invitrogen), and CD62L (Clone MEL 14, BD Biosciences, Waltham, MA, USA). Prior to staining for the transcription factor Ki-67 (clone SolA15; eBioscience), the cells were permeabilized using the Foxp3/Transcription Factor Staining Buffer Set (eBioscience) for 30 min at 4 °C.

To determine the activation of macrophages, dendritic cells, and B and T cells, the isolated splenocytes and lymphocytes were blocked using CD16/CD32 (eBioscience; Thermo Fisher Scientific Inc.) for 20 min at 4 °C, and were stained using Fixable Viability Dye eFluor520 (eBioscience), F4/80 (clone BM8, eBioscience), CD11c (clone N418, eBioscience), CD11b (clone M1/70, eBioscience), Ova-FITC (Thermo Fisher Scientific), CD40 (clone 1C10, eBioscience), CD80 (clone 16-10A1, Biolegend, San Diego, CA, USA), CD86 (clone GL1, Biolegend), XCR1 (clone ZET, Biolegend), CD8a (clone 53–6.7, eBioscience), CD19 (clone 6D5, Biolegend), B220 (clone RA3-6B2, BD Biosciences), CD4 (clone GK1.5, Invitrogen), and CD8a (clone 53–6.7, Invitrogen) for 20 min at 4 °C without exposure to light. Stained cells were fixed with 1% paraformaldehyde. Cells were analyzed using the FACS Aurora (Cytek Biosciences, Fremont, CA, USA), CytoFLEX Flow Cytometer (Beckman Coulter, CA, USA), and the data were analyzed using Spectroflo (Cytek Biosciences), FlowJo (Tree Star, Inc., Ashland, OR, USA), and CytExpert (Beckman Coulter).

### Splenocyte cell culture and Enzyme-Linked ImmunoSpot (ELISpot)

Splenocytes were stimulated with 4 µg/well Ova 323–339 CD4-specific T cell (ISQAVHAAHAEINEAGR) and Ova 257–264 CD8-specific T cell epitope peptide (SIINFEKL) for 48 h at 37 °C. The OVA peptides were synthesized using Peptron (Daejeon, Korea). The ELISPOT assays for the detection of IFN-γ-secreting T cells were performed using mouse IFN-γ (Mabtech, Stockholm, Sweden), according to the manufacturer’s instructions.

### Preparation of bone marrow-derived dendritic cells (BMDCs)

The cells were prepared as previously described [42]. Bone marrow nucleated cells (1 × 10^6^ cells/ml) were seeded in a modified 5 ml RPMI 1640 (Gibco; Thermo Fisher Scientific Inc.), Waltham, MA, USA) medium containing 10% FBS (Gibco), in 6-well plates. Subsequently, 100 ng/ml mouse recombinant GM-CSF (Granulocyte–Macrophage Colony-Stimulating Factor) (Peprotech, New Jersey, United States) and 50 ng/ml mouse recombinant IL-4 (Peprotech) were added to the medium to stimulate BMDCs.

### Mouse cancer challenge

B16-Ova cells (5 × 10^5^ cells) were subcutaneously injected into 6-week-old female C57BL/6 mice on day 0. On day 7 after tumor inoculation, tumor size was measured and the mice were intramuscularly immunized with one of the following: Ova protein (50 µg) in isolation, Ova protein (50 µg) in combination with poly(I:C) (20 µg), or Ova protein (50 µg) in combination with ssRNA (20 µg), at an interval of 2 days (days 9, 11, 13, and 15) to total 5 immunization treatments (*n* = 5). The mice were sacrificed when the size of tumor exceeded 1 cm^3^ according to animal ethical standards. On day 31 after the tumor challenge, the weight of the tumors from the sacrificed mice was measured.

### Statistical analyses

All data are expressed as the mean ± SD. One-way ANOVA was used to assess the significance of differences among treatment groups; if significant deviations from variance homogeneity were detected using the Levene test, then the nonparametric Kruskal–Wallis H test was performed. The student’s *t*-test was used to assess significant differences between the two groups; if significant deviations from variance homogeneity were detected using the Levene test, then the nonparametric Mann–Whitney U test was performed. Statistical analyses were conducted using SPSS for Windows (release 14.0 K, IBM, Armonk, NY). Differences were considered significant at values of *p < 0.05, **p < 0.01, and ***p < 0.005.

## Results

### CrPV^IRES^-ssRNA recruits immune cells from the inguinal lymph node

The immune response induced by CrPV^IRES^-ssRNA was assessed using poly(I:C) and alum as positive controls. The immune cells in the drain inguinal lymph nodes were counted at an earlier time point (Fig. 1A, B). The CrPV^IRES^-ssRNA treated group demonstrated an increase in the number of cells isolated from drain inguinal lymph nodes 24 h after immunization (Fig. 1A, B; Supplementary Fig. S1A). The Poly(I:C) treatment group showed a similar increase in cells isolated from the drain inguinal lymph nodes, while no increase in immune cells was observed in the alum treated group (Fig. 1B). However, the proportion of CD4^+^/CD8^+^ T cells in drain lymph nodes did not change significantly in any of the groups (Supplementary Fig. S1B, C). Subsequently, the antigen uptake ability of activated APCs was evaluated using the fluorescein isothiocyanate-conjugated Ova (Ova-FITC). The antigen uptake ability was significantly higher in the CrPV^IRES^-ssRNA and poly(I:C) treated group than in the alum treated group (Fig. 1C, D). This indicates that CrPV^IRES^-ssRNA can act as an adjuvant by effectively recruiting antigen-presenting cells and enhancing antigen uptake.

### CrPV^IRES^-ssRNA activates dendritic cells in drain inguinal lymph nodes

To identify the types of APCs recruited by CrPV^IRES^-ssRNA treatment, activation markers of dendritic cells and macrophages, such as CD40^+^, CD80^+^, and CD86^+^, were measured by flow cytometry. Bone marrow-derived dendritic cells (BMDCs) were treated with CrPV^IRES^-ssRNA and poly(I:C). The CrPV^IRES^-ssRNA treatment increased levels of the activation markers in a dose-dependent manner (Fig. 2A; Supplementary Fig. S2A). After we injected poly(I:C) and CrPV^IRES^-ssRNA intramuscularly without the antigen, inguinal lymph nodes were collected on day 1 (Fig. 2B). In the CrPV^IRES^-ssRNA-treated group, the expression of the co-stimulatory molecules, CD40^+^, CD80^+^, and CD86^+^, increased in CD11c-positive cells related to dendritic cells regardless of the concentration (Fig. 2C). However, the expression of macrophage activation markers in poly(I:C)- and CrPV^IRES^-ssRNA-treated groups was not significantly different from that in the control group (Supplementary Fig. S2B). Next, we investigated the dendritic cell types of drain lymph nodes affected by uptake of CrPV^IRES^-ssRNA treatment. The activated conventional DC1/2 (cDC1 and cDC2) and pDC in drain inguinal lymph nodes were identified using flow cytometry. The CrPV^IRES^-ssRNA treatment enhanced the activity of the CD8a^+^ cDC1 activation maker, while differences in the CD8a^−^ cDC2 and pDC markers were insignificant (Fig. 2D, E). The poly(I:C)-treated group also showed a similar pattern (Fig. 2D-E). These results revealed that CrPV^IRES^-ssRNA primarily increases the activity of CD8a^+^ cDC1 cells among antigen-presenting cells.

### Ovalbumin (Ova) protein formulated with CrPV^IRES^-ssRNA activates CD8a.^+^ cDC1

In immunology, Ova is widely used in vaccines as it is a key protein that stimulates immune responses, including allergic reactions [43]. To investigate the detailed mechanism of cDC1 activation by CrPV^IRES^-ssRNA treatment, we formulated a combination of CrPV^IRES^-ssRNA or poly(I:C) with Ova protein. After the second immunization (day 0 and day 14) with these constructs in mice, splenocytes and inguinal lymph nodes lymphocytes were harvested after one day (day 14 + 1) (Fig. 3A). Treatment of CrPV^IRES^-ssRNA with Ova protein resulted in enhanced expression of the co-stimulatory molecules, CD40^+^, CD80^+^, and CD86^+^, on CD8a^+^DC1 cell population in spleen and inguinal lymph nodes (Fig. 3B, C). Additionally, an increased expression of the chemokine receptor, XCR1^+^, which is also an activation marker of CD8a^+^ DC1 cells, was observed in the spleen and inguinal lymph node cells (Fig. 3D). This suggested that CrPV^IRES^-ssRNA with Ova protein could act as an adjuvant therapy by activating CD8a^+^ cDC1 cells.

### CrPV^IRES^-ssRNA formulated with Ova protein elicits production and activation of T cells

To evaluate CrPV^IRES^-ssRNA-mediated T-cell activation, splenocytes, and inguinal lymph nodes lymphocytes were isolated on day 7 after the second immunization (day 14 + 7) of mice with saline (Group 1; G1), Ova protein (Group 2; G2), Ova with alum (Group 3; G3), and CrPV^IRES^-ssRNA formulated with Ova (Group 4; G4) (Fig. 4A). The population of Ova-specific CD8^+^ T cells in the spleen significantly increased in the CrPV^IRES^-ssRNA-Ova-treated group (G4) (Fig. 4B, C). Furthermore, G4 showed statistically significant difference increased population of Ova-specific CD8 effector T cells and Ki-67^+^, a proliferation marker, compared to G2 in the drain inguinal lymph nodes (Fig. 4D). IFN-γ is an inflammatory cytokine with anti-tumor function that is related to innate and adaptive immune responses [44]. In the CrPV^IRES^-ssRNA-Ova-treated group, the frequency of IFN-γ-secreting cells after stimulation with CD4 and CD8 specific peptides was significantly higher than that of G2 and G3 (Fig. 4E). These data suggest that CrPV^IRES^-ssRNA treatment enhances dendritic cell uptake and activates CD8a^+^ cDC1, especially when administered alone or in combination with antigen, leading to activated antigen-specific CD8 T cells.

### CrPV^IRES^-ssRNA reduces tumor burden in a murine melanoma model

**Fig. 5:**
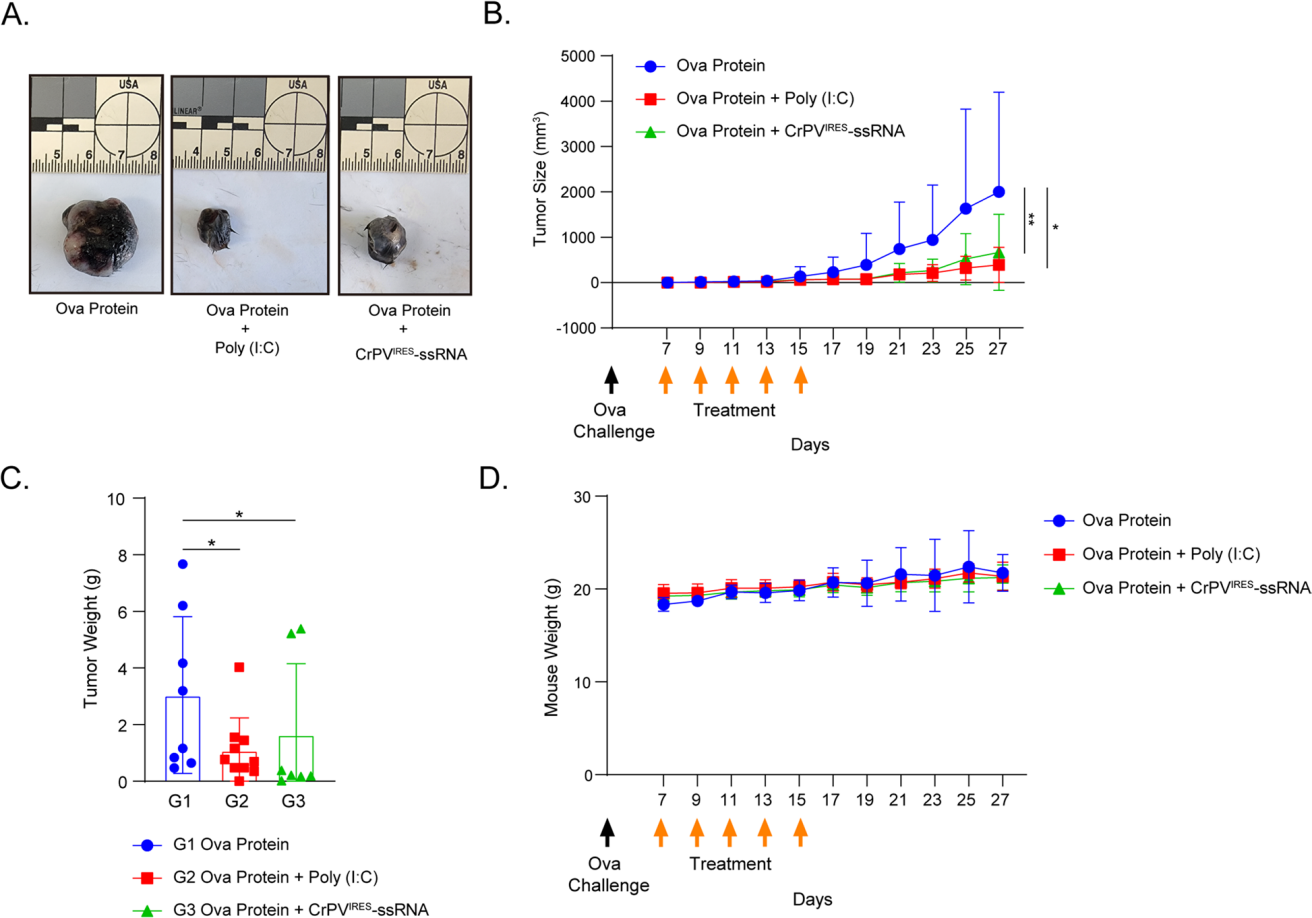
CrPV^IRES^-ssRNA reduces tumor burden in mouse melanoma. Mice (C57BL/6) were challenged with a subcutaneous injection of B16-Ova cells (5 × 10.^5^ cells) on day 0. Then, the mice were immunized intramuscularly with Ova protein (50 μg), poly(I:C) (20 μg) and CrPVIRES-ssRNA (20 μg) every 2 days until day 7. **A** Representative tumor images harvested on day 31. **B** Tumor size was measured every 2 days in the indicated groups. **C** Weight of tumors harvested on day 31. **D** Mice weights were measured every 2 days. Data were represented as mean ± SD. Statistical significance was indicated by *p < 0.05 and **p < 0.01

**Fig. 6:**
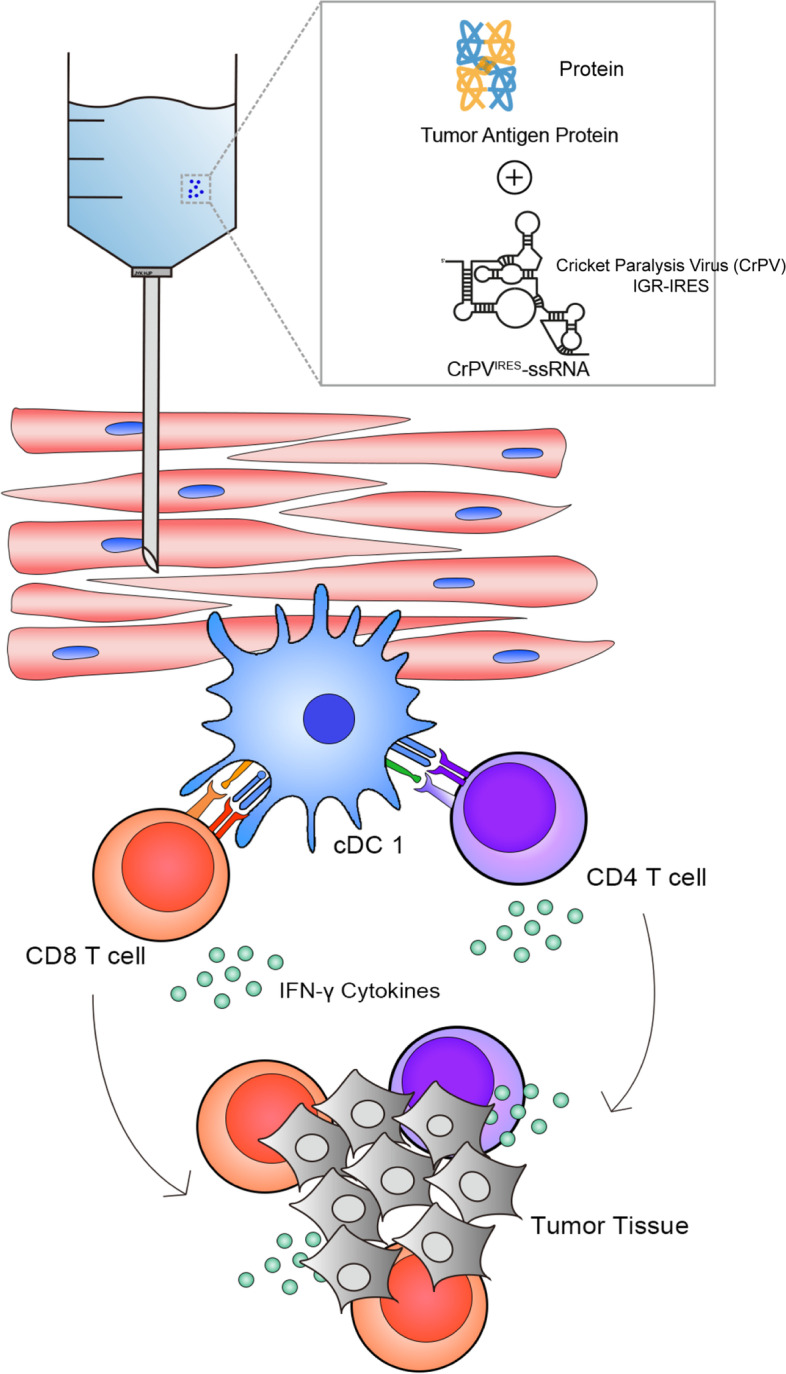
CrPVIRES-ssRNA activates immune signaling pathways associated with reduced mouse melanoma

To test the *in-vivo* efficacy of CrPV^IRES^-ssRNA, mice were challenged with B16-Ova cells. Tumor-bearing mice (ten in each group) were inoculated intramuscularly with Ova protein, Ova protein-poly(I:C), and Ova protein-CrPV^IRES^-ssRNA. In the poly(I:C)- and CrPV^IRES^-ssRNA-treated groups, there was a decrease in cancer growth and weight compared to the control (Ova protein-treated) group (Fig. 5A-C). Mouse weight was not significantly different between groups (Fig. 5D). In addition, CrPV^IRES^-ssRNA and poly(I:C) treatment increased the survival rate. (Supplementary Fig. S3A). These results suggest that immune cells augmented by CrPV^IRES^-ssRNA may exhibit potential anti-tumor effects by protecting against melanoma growth (Fig. 6).

## Discussion

Cancer immunotherapy has been actively studied as a promising strategy in cancer treatment. However, there are many limitations in treating cancer such as immune tolerance and insufficient immune response. Adjuvant therapies that increase the immune response may play an important role in overcoming these limitations. For instance, cancer vaccines that introduce tumor antigens to APCs can enhance anti-tumor immune responses by attenuating immune resistance and generating sufficient immune responses in the cancer environment [45]. Dendritic cells, a type of APC activated by cancer vaccines, can initiate immune cell migration and activation [46], leading to the activation of antigen-specific CD4/CD8 T cells that can directly attack cancer.

Previous studies have reported that double-stranded RNA may be an important substance in cancer vaccines and may itself increase immune responses via TLR3 and RIG-Is [47]. Previous findings have confirmed that the CrPV^IRES^-ssRNA increased the expression of CXCR1, an inflammatory dendritic cell marker, around the injection site [35, 48]. In this study, we investigated the mechanism of the novel viral IRES-derived CrPV^IRES^-ssRNA in inducing immune responses [35]. CrPV^IRES^-ssRNA gradually increased the total cell population such as DCs, macrophages, and B cells in the drain inguinal lymph nodes. Moreover, it enhanced the activation of APCs (Fig. 1). In general, activated APCs have been reported to enhance antigen presentation to major histocompatibility (MHC) molecules, leading to adaptive and innate immunity [12, 49, 50]. Expression analysis of co-inflammatory signature proteins revealed that CrPV^IRES^-ssRNA increased antigen uptake by dendritic cells. Notably, CrPV^IRES^-ssRNA treatment significantly increased the co-stimulatory molecules of CD8^+^cDC1, such as CD40^+^, CD80^+^, CD86^+^, and XCR1^+^ (Figs. 2 and 3), suggesting that CrPV^IRES^-ssRNA is strongly associated with specific types of DCs. It is known that CD8^+^ cDC1 cells contribute to the effective cross-presentation of antigens [51–53] and the induction of Th1 responses [54, 55], as well as enhancing the production of chemokines associated with effector and memory CTLs [56, 57]. Functionally, expression of XCR1^+^ on CD8^+^ cDC1 leads to an increase in the proliferation and activation of antigen-specific CD8^+^ T cells and secretion of IFN-γ [58, 59]. These results indicated that CrPV^IRES^-ssRNA can induce a general immune response by stimulating APCs, especially cDC1 cells, to induce activation of effector T cells.

In addition, this study investigated whether CrPV^IRES^-ssRNA induces antigen-specific T-cell responses. CrPV^IRES^-ssRNA significantly increased the total population and proliferative capacity of antigen-specific effector CD8 T cells in drain inguinal lymph nodes (Fig. 4D), suggesting that CrPV^IRES^-ssRNA activates antigen-specific CD8 T cells by stimulating the maturation of cDC1 cells. It has been reported that cDC 1 cells induce the expansion of CD8 T cells primarily by presenting specific antigens expressed on MHC class I molecules [60]. The activation of CD8 T cells was involved in anti-tumor immune responses [61, 62] and anti-viral responses [63]. Interestingly, CrPV^IRES^-ssRNA increased the frequency of IFN-γ-secreting cells (Fig. 4E). In addition, although not statistically significant in the spleen of challenged mice, a slight increase the population of IFN-γ-secreting cells can be seen than in the negative control group (G1) (Supplementary Fig. S3B). It has been observed that antigen-specific CD4 and CD8 T cells inhibited the growth of melanoma cells, mainly by inducing IFN-γ cytokines that increase tumor-infiltrating lymphocytes and activating APCs to kill melanoma cells [64]. Indeed, CrPV^IRES^-ssRNA delayed the growth of cancer cells in a murine melanoma model (Fig. 5).

## Conclusions

In conclusion, a novel cricket paralysis virus IRES-based single-stranded RNA may play an essential role as an adjuvant in inducing cDC1-CD8 T cell-mediated immune responses in cancer immunotherapy. Based on the immunological mechanism induced by the ssRNA adjuvant (Fig. 6), it can be applied to various cancer vaccines and adjuvant cancer treatment regimes.

## Supplementary Information

**Addition al file 1: Supplementary Fig. S1. **CrPV^IRES^-ssRNA recruits immune cells into drain lymph node. C57BL/6 mice were intramuscularly injected with alum, CrPV^IRES^-ssRNA, and poly(I:C) for 48 h. Macrophage and T cells were analyzed using flow cytometry in drain inguinal lymph nodes. (A) Cell percentage of macrophages in the indicated groups (*n* = 3). (B, C) Cell percentage of CD4-positive T cells (B) and CD8-positive T cells (C) in drain inguinal lymph nodes (*n* = 3). Data were represented as mean ± SD. Statistical significance was indicated by *p < 0.05.

**Additional file 2: Supplementary Fig. S2.**CrPV^IRES^-ssRNA activates co-stimulatory molecules, CD40, CD80, and CD86. (A) BMDCs were stimulated with CrPV^IRES^-ssRNA or poly(I:C) according to the table for 6 h. Cell activation was measured by flow cytometry using antibodies to CD40, CD80, and CD86. (B) The inguinal lymph nodes were isolated 1 day after immunization. Plots show percentages of co-stimulatory molecules CD40, CD80, and CD86 of macrophages in lymphocytes isolated from drain inguinal lymph nodes. Data were represented as mean ± SD. Statistical significance was indicated by *p < 0.05, **p < 0.01, and ***p < 0.005.

**Additional file 3: Supplementary Fig. S3. **CrPV^IRES^-ssRNA is associated with melanoma antigen specific IFN- γ. After challenge with a subcutaneous injection of B16-Ova cells (5×10^5^cells), the mice (C57BL/6) were immunized intramuscularly with Ova protein (50 μg), poly(I:C) (20 μg), and CrPV^IRES^-ssRNA (20 μg) every 2 days until day 7. (A) According to animal ethics standards, mice were sacrificed when the size of the cancer was greater than 1 cm^3^.The survival % was measured for mice in all groups over time to day 27 (*n *= 10). (B) Splenocytes were stimulated for 2 days with/without Ova-specific CD4 or CD8 T cell peptide. The IFN-γ-producing cells were measured using ELISpot in the indicated groups.

## Data Availability

All data generated or analyzed during this study are included in this published article and its supplementary information files.

## Abbreviations

ssRNA: Single-stranded RNA
CrPV: Cricket paralysis virus
IRES: Internal ribosome entry site
cDC1: Conventional dendritic cell 1
PRRs: Pattern recognition receptors
TLRs: Toll-like receptors
RIG-I: Retinoic acid-inducible gene I
RIG-Is: RIG-I-like receptors
APCs: Antigen-presenting cells
cDCs: Conventional dendritic cells
pDCs: Plasmacytoid dendritic cells
UV: Ultraviolet
Ova-FITC: Fluorescein isothiocyanate-conjugated Ova
cDC1,cDC2: Conventional DC1/2
