# Supplementary figures and images for: Adjuvant effect of IRES-based single-stranded RNA on melanoma immunotherapy
Source: BMC Cancer. 2022 Oct 5;22:1041. doi: 10.1186/s12885-022-10140-2 (PMC9533600; doi:10.1186/s12885-022-10140-2)

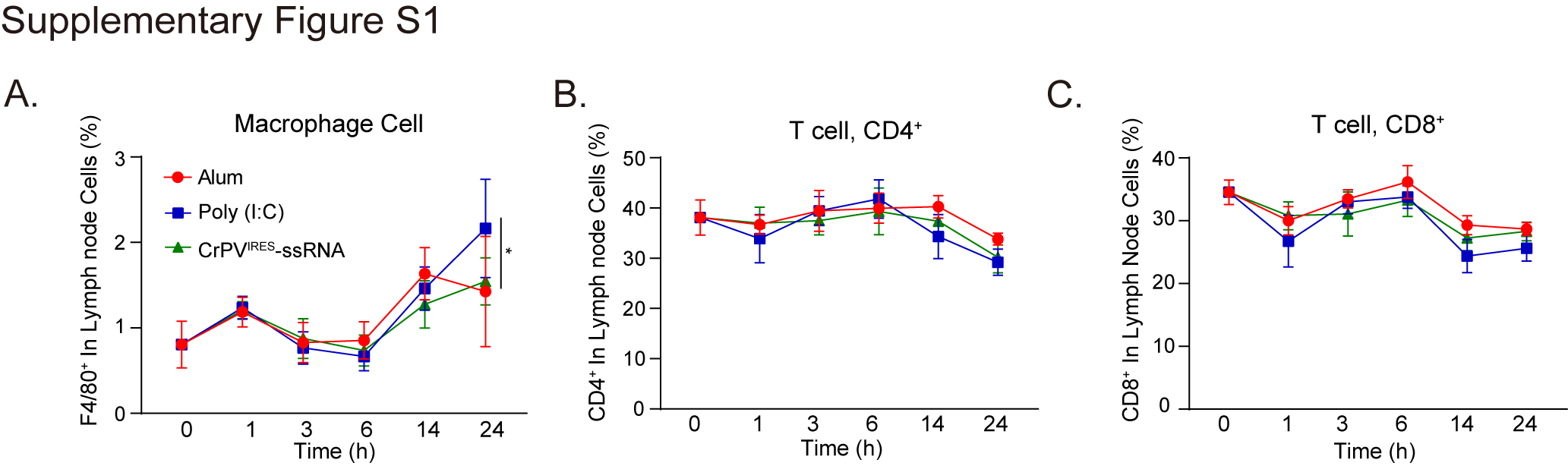

Supplement: Supplementary file 1 — Addition al file 1: Supplementary Fig. S1. CrPVIRES-ssRNA recruits immune cells into drain lymph node. C57BL/6 mice were intramuscularly injected with alum, CrPVIRES-ssRNA, and poly(I:C) for 48 h. Macrophage and T cells were analyzed using flow cytometry in drain inguinal lymph nodes. (A) Cell percentage of macrophages in the indicated groups (n = 3). (B, C) Cell percentage of CD4-positive T cells (B) and CD8-positive T cells (C) in drain inguinal lymph nodes (n = 3). Data were represented as mean ± SD. Statistical significance was indicated by *p < 0.05. [file 12885_2022_10140_MOESM1_ESM.tif]

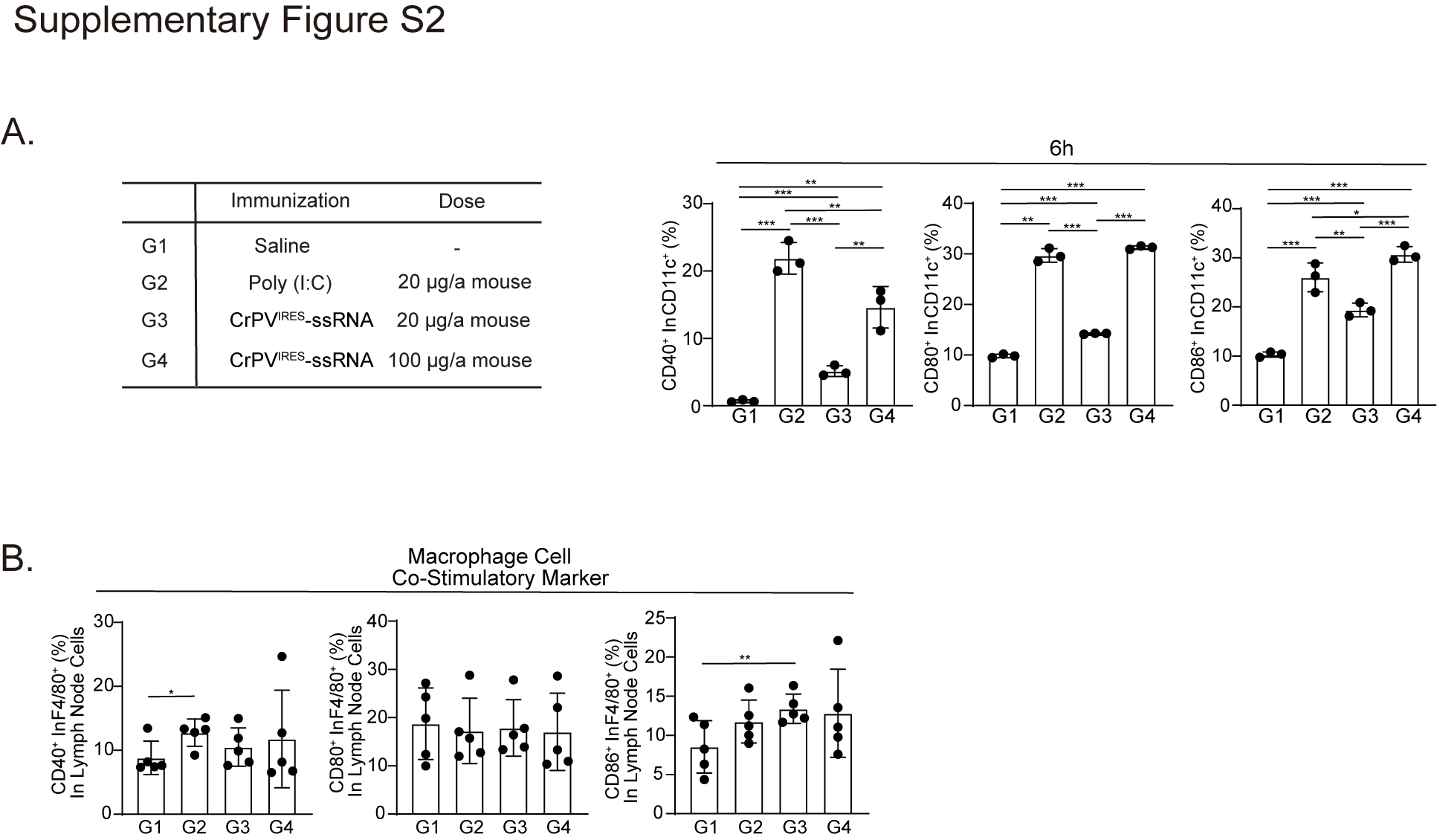

Supplement: Supplementary file 2 — Additional file 2: Supplementary Fig. S2.CrPVIRES-ssRNA activates co-stimulatory molecules, CD40, CD80, and CD86. (A) BMDCs were stimulated with CrPVIRES-ssRNA or poly(I:C) according to the table for 6 h. Cell activation was measured by flow cytometry using antibodies to CD40, CD80, and CD86. (B) The inguinal lymph nodes were isolated 1 day after immunization. Plots show percentages of co-stimulatory molecules CD40, CD80, and CD86 of macrophages in lymphocytes isolated from drain inguinal lymph nodes. Data were represented as mean ± SD. Statistical significance was indicated by *p < 0.05, **p < 0.01, and ***p < 0.005. [file 12885_2022_10140_MOESM2_ESM.tif]

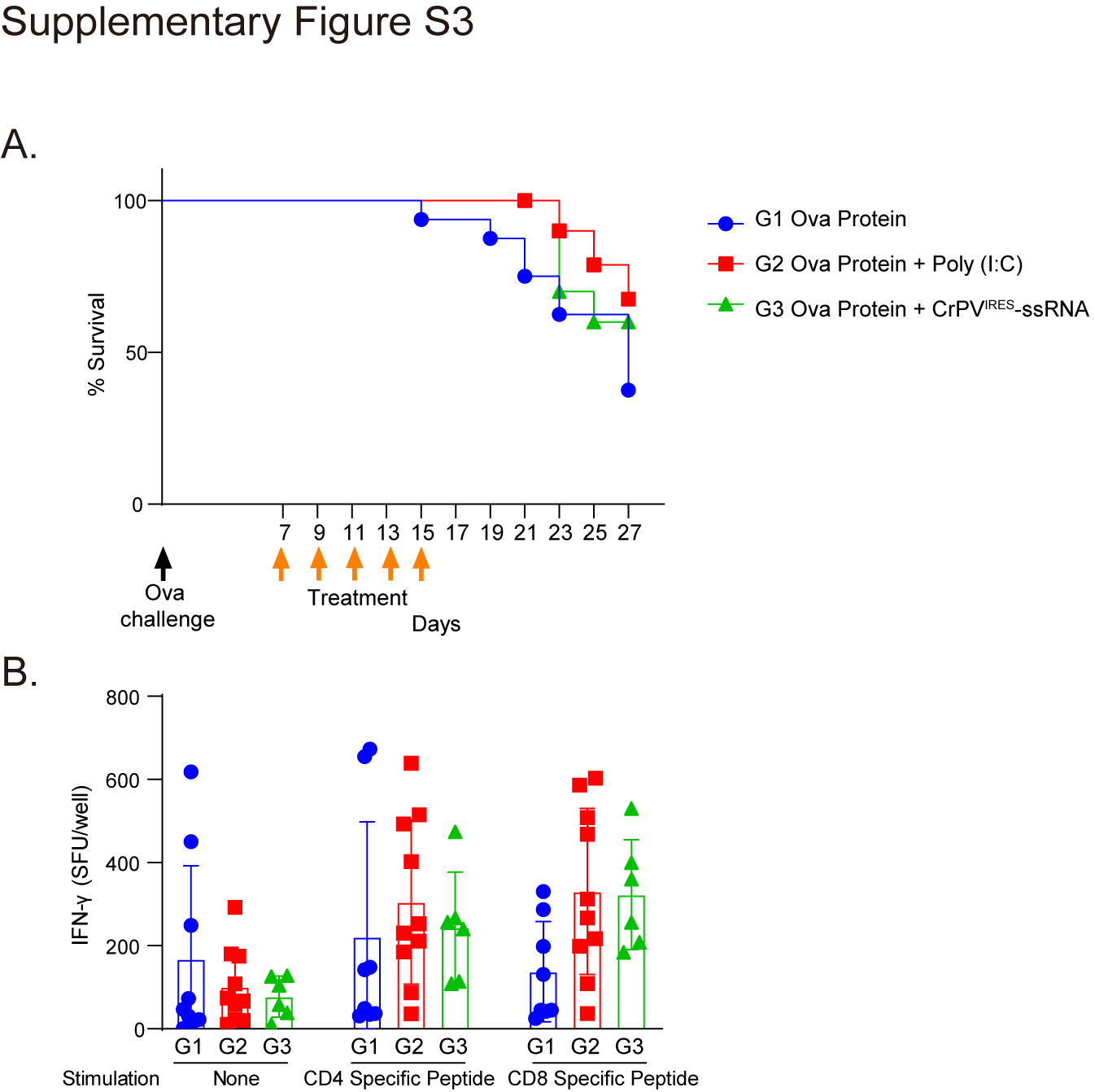

Supplement: Supplementary file 3 — Additional file 3: Supplementary Fig. S3. CrPVIRES-ssRNA is associated with melanoma antigen specific IFN- γ. After challenge with a subcutaneous injection of B16-Ova cells (5×10^5^cells), the mice (C57BL/6) were immunized intramuscularly with Ova protein (50 μg), poly(I:C) (20 μg), and CrPVIRES-ssRNA (20 μg) every 2 days until day 7. (A) According to animal ethics standards, mice were sacrificed when the size of the cancer was greater than 1 cm3.The survival % was measured for mice in all groups over time to day 27 (n = 10). (B) Splenocytes were stimulated for 2 days with/without Ova-specific CD4 or CD8 T cell peptide. The IFN-γ-producing cells were measured using ELISpot in the indicated groups. [file 12885_2022_10140_MOESM3_ESM.tif]
